# Prediction of COVID‐19 patients in danger of death using radiomic features of portable chest radiographs

**DOI:** 10.1002/jmrs.631

**Published:** 2022-11-05

**Authors:** Maoko Nakashima, Yoshikazu Uchiyama, Hirotake Minami, Satoshi Kasai

**Affiliations:** ^1^ Graduate School of Health Sciences Kumamoto University Kumamoto Japan; ^2^ Department of Medical Image Sciences, Faculty of Life Sciences Kumamoto University Kumamoto Japan; ^3^ Konica Minolta, Inc. Tokyo Japan; ^4^ Department of Radiological Technology Niigata University of Health and Welfare Niigata Japan

**Keywords:** Artificial intelligence, COVID‐19, portable chest X‐ray, prognosis prediction, radiomics

## Abstract

**Introduction:**

Computer‐aided diagnostic systems have been developed for the detection and differential diagnosis of coronavirus disease 2019 (COVID‐19) pneumonia using imaging studies to characterise a patient's current condition. In this radiomic study, we propose a system for predicting COVID‐19 patients in danger of death using portable chest X‐ray images.

**Methods:**

In this retrospective study*,* we selected 100 patients, including ten that died and 90 that recovered from the COVID‐19‐AR database of the Cancer Imaging Archive. Since it can be difficult to analyse portable chest X‐ray images of patients with COVID‐19 because bone components overlap with the abnormal patterns of this disease, we employed a bone‐suppression technique during pre‐processing. A total of 620 radiomic features were measured in the left and right lung regions, and four radiomic features were selected using the least absolute shrinkage and selection operator technique. We distinguished death from recovery cases using a linear discriminant analysis (LDA) and a support vector machine (SVM). The leave‐one‐out method was used to train and test the classifiers, and the area under the receiver‐operating characteristic curve (AUC) was used to evaluate discriminative performance.

**Results:**

The AUCs for LDA and SVM were 0.756 and 0.959, respectively. The discriminative performance was improved when the bone‐suppression technique was employed. When the SVM was used, the sensitivity for predicting disease severity was 90.9% (9/10), and the specificity was 95.6% (86/90).

**Conclusions:**

We believe that the radiomic features of portable chest X‐ray images can predict COVID‐19 patients in danger of death.

## Graphical abstract

We developed an artificial intelligence approach capable of identifying patients at higher risk of developing more severe COVID‐19 by using radiomic features obtained from portable chest x‐ray images. Our approach may have important clinical relevance during the pandemic.

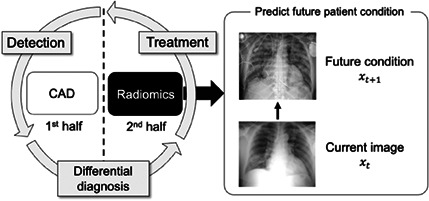

## Introduction

The number of people who have been infected with coronavirus disease (COVID‐19) now exceeds 277 million worldwide, with approximately 5.3 million deaths. Early detection and prevention of severe disease are both important for COVID‐19 infection control, and artificial intelligence (AI) has been used to achieve this goal.

**Figure 1:**
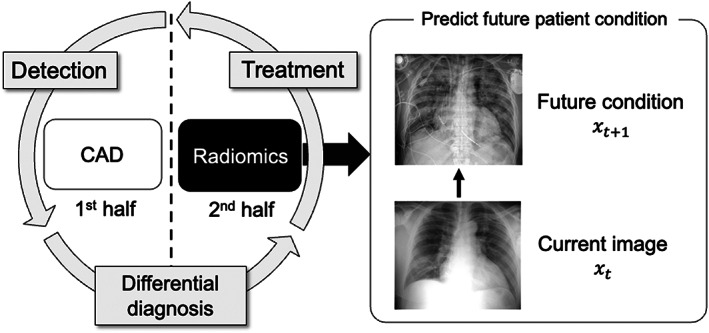
Difference between computer‐aided diagnosis (CAD) and radiomic research.

Computer‐aided diagnosis (CAD) and radiomics have been used to develop AI systems in radiology. CAD systems support the detection and differential diagnoses of various diseases, including breast, lung and colon cancers.^1^, ^2^, ^3^ These systems estimate the likelihood of a disease state from current images. In comparison, radiomics support medical care after a disease is detected^4^ by predicting prognosis or therapeutic effects. Therefore, radiomics differ from CAD because it predicts a future state from a current image. In general, medical care is performed in the following order: disease detection, differential diagnosis and treatment. Therefore, CAD can be considered an AI strategy that supports the first half of medical care, while radiomics support the second half of medical care, as shown in Figure 1.

Research related to COVID‐19 involves both CAD and radiomic studies. CAD research includes studies related to the detection of COVID‐19 pneumonia,^5^ the differentiation of COVID‐19 pneumonia from other types of pneumonia,^6^, ^7^ the concurrent detection and differential diagnosis of COVID‐19 pneumonia^8^, ^9^, ^10^, ^11^, ^12^, ^13^ and the differential diagnosis of severe respiratory failure.^14^ On the other hand, radiomic research includes predictive studies related to the severity and prognosis of COVID‐19,^15^, ^16^, ^17^, ^18^, ^19^, ^20^, ^21^ the need for oxygenation support and intubation^22^ and the criteria for discharge.^23^, ^24^

In previous radiomic studies of COVID‐19, computed tomography (CT) images were used because of the ease of analysis, while chest radiographs have never been used. However, preventing infection during entering and exiting the CT room imposes a heavy burden on the medical staff. Therefore, in this study, we developed a method for predicting COVID‐19 patients in danger of death using portable chest radiographs that can be obtained in a hospital room. However, since chest radiography is a two‐dimensional projection of a three‐dimensional lung structure, images can be difficult to analyse because of overlapping bone components and lesions. In this study, we analysed COVID‐19 lesion patterns after image pre‐processing to attenuate bone components. We then analysed the usefulness of this approach by comparing the predictive performance of our system with and without this bone‐suppression technique.

## Materials and Methods

### Image and clinical data

For this study, we used the COVID‐19‐AR database of the Cancer Imaging Archive,^25^ which contains clinical and imaging data from 105 patients with severe acute respiratory syndrome coronavirus 2 infection. We selected 100 portable chest radiographs taken when patients were first hospitalised and used them for the analyses in this study. Patients with cardiac pacemakers were excluded. Ten patients who died after treatment and 90 patients who recovered were selected for this study. Approval from the ethics review committee of Kumamoto University was obtained for the implementation of this study.

### Bone‐suppression technique

**Figure 2:**
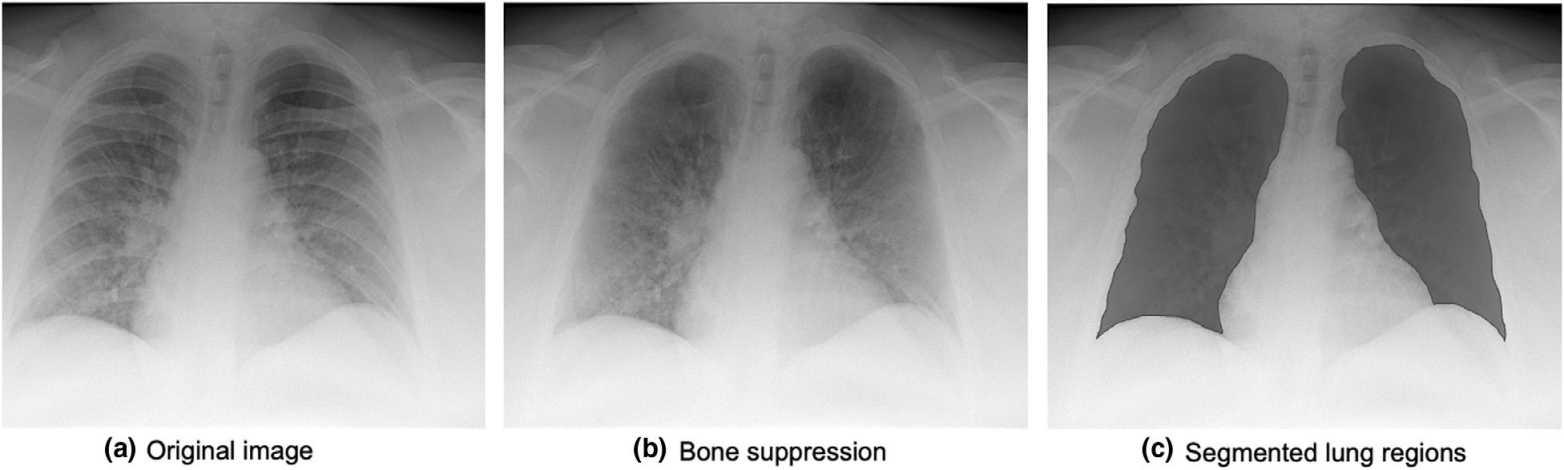
Examples of images with bone suppression and manually segmented lung regions.

Reticular and ground‐glass opacities have been detected on CT images of COVID‐19 patients.^26^, ^27^ However, using portable chest radiographs to detect these shadows can be difficult because of bone overlap with bone. Therefore, a bone‐suppression technique,^28^ developed by Konica Minolta, was employed in this study. Figure 2B shows an example of an output image with bone suppression. This process was performed using the following three steps: first, the lung area was classified into four regions with different boundary properties, including the lung apex, external thoracic region, diaphragm and mediastinum. Each lung region was then extracted using the optimal edges for each boundary. Next, edges and ridges related to clavicles and ribs were detected with the use of the first and second derivatives of specific direction on the image, and then, boundaries of ribs and clavicles were extracted with the use of the bone model extracted from chest image database, which was consisted of bone edge angle and location. Once bone edges were identified, signal distribution from clavicles and ribs were estimated and distinguished from background signal to suppress the signal from bones. During the signal estimation process, signal distribution from clavicles and ribs was estimated in a cross‐sectional direction with the use of Gaussian smoothing and morphometric technologies, and suppressed from the image. Finally, clavicles and posterior and anterior ribs were suppressed.^28^

### Determination of radiomic features

In this study, the left and right lung regions, excluding the diaphragm and heart, were manually segmented, and radiomic features were determined from each segmented lung region. Figure 2C shows an example of a segmented lung region. The diaphragm and heart were excluded because COVID‐19 lesions are usually detected in the peripheral lung regions.^26^, ^27^ In addition, the high pixel values in the normal tissues of the diaphragm and heart can overlap with the radiomic features of COVID‐19 lesions in the lung region.

In this study, 310 radiomic features were calculated from each of the segmented regions of the left and right lungs, for a total of 620 radiomic features. The free software MaZda^29^, ^30^, ^31^ was used to calculate these features. The 310 radiomic features in each lung included a size feature, nine histogram features, 272 texture features and 28 resolution features. The default values of MaZda were adopted as parameters for calculating these radiomic features. For example, the parameters used for calculating the density co‐occurrence matrix of the texture features were 16 density gradations, a distance between 1 and 5 pixels, and 0°, 45°, 90° and 135° in the direction.

### Selection of radiomic features

The number of radiomic features was 610, which was larger than the 100 cases. Hence, it was necessary to identify radiomic features that were useful for the prediction of COVID‐19 patients in danger of death. In this study, radiomic features were selected using the least absolute shrinkage and selection operator (Lasso) technique,^32^ which was defined by the following equation:

(1) $${\hat{\beta }}^{\text{lasso}}=\underset{\beta }{\text{argmin}}\left\{\frac{1}{2}\sum_{i=1}^{N}{\left(\left.y_{i}-\beta_{0}-\sum_{j=1}^{p}x_{\mathrm{ij}}\beta_{j}\right)\right.}^{2}+\lambda \sum_{j=1}^{p}\left.\left|\beta_{j}\right.\right|\right\}.$$

Here, $y_{i}$ was the prognostic information, i.e. the death or recovery of the i‐th patient. $x_{j}$ was the radiomic feature; $\beta_{j}$ was the coefficient; and $\beta_{0}$ was the constant term. λ≥0 was a complexity parameter that controlled the degree of reduction, and p represented the total number of radiomic features. The parameter $\beta_{j}$ was obtained by solving the quadratic programming problem in equation (1). In this study, λ was set so that the number of radiomic features with the non‐zero coefficient $\beta_{j}$ was 4. Three‐fold cross‐validation was then performed to determine the value of λ that minimised the average deviation. When the values of λ obtained in the process of this calculation were used in order, the value of λ was adopted so that the number of radiomic features with non‐zero coefficients was 4.

### Visualisation of multidimensional scaling

Although Lasso can reduce the dimensions of radiomic features, the resultant data are still multidimensional. Therefore, it can be difficult to understand the relationship between the multidimensional data and the patients in danger of death. If, however, the data can be reduced to two dimensions, this relationship can be visualised as a scatter plot. Therefore, we employed multidimensional scaling (MDS)^32^ to reduce radiomic features to two dimensions. MDS was used to construct a new axis via the following procedure: first, the distance matrix $d_{\mathrm{ij}}$ consisting of the Euclidean distance of input i and input j was calculated, and then, the transformation matrix $z_{\mathrm{ij}}$ was calculated using the following equation:

(2) $$z_{\mathrm{ij}}=-\frac{1}{2}\left(d_{\mathrm{ij}}^{2}-\sum_{i=1}^{n}\frac{d_{\mathrm{ij}}^{2}}{n}-\sum_{j=1}^{n}\frac{d_{\mathrm{ij}}^{2}}{n}+\sum_{i=1}^{n}\sum_{j=1}^{n}\frac{d_{\mathrm{ij}}^{2}}{n^{2}}\right).$$

The transformation matrix moved the origin to the center of gravity of the n input data. Finally, the new coordinate points were determined as coordinate values on the axis given by the eigenvector of the transformation matrix $z_{\mathrm{ij}}$. Because MDS is a linear transformation that maintains the Euclidean distance between data, it can be interpreted to reproduce the relative positional relationship of multidimensional data in a low‐dimensional space.

### Prediction of patients in danger of death

We employed classifiers from four radiomic features selected by Lasso to predict death and recovery cases. Linear discriminant analysis (LDA)^33^, ^34^ and an SVM^35^ were used in this study. LDA is a method for determining the hyperplane that best discriminates between death and recovery cases when the variance in each group is assumed to be the same in the feature space. Unfortunately, a high discriminative performance cannot be obtained with LDA when the input data contains ‘outliers’. However, when LDA provides high discriminative performance, it signifies that there is a simple relationship between radiomic features and the mortality risk of COVID‐19. Therefore, if radiologists can interpret imaging findings that match the radiomic features, it may be possible to predict the mortality risk of COVID‐19.

On the other hand, an SVM can generate a decision boundary when outliers are removed by a technique called soft margin. In addition, kernel tricks can generate complex decision boundaries, which enables highly accurate discrimination, even for problems that cannot be linearly separated. By comparing the discriminative performance of LDA with SVM, it is possible to understand the complexity of the overlap in the multidimensional radiomic feature space for the two groups (that is, death and recovery groups). In this experiment, a Gaussian kernel with σ = 3 and a soft margin C = 0.01 was employed. These parameters were empirically determined. For training and testing these classifiers, the leave‐one‐out method^33^, ^34^ was employed. To evaluate the discriminative performance, the area under the curve (AUC) of the receiver‐operating characteristic (ROC) analysis was used. The LABROC4^36^ algorithm developed by the University of Chicago was used for the ROC analysis.

## Experimental Results

**Figure 3:**
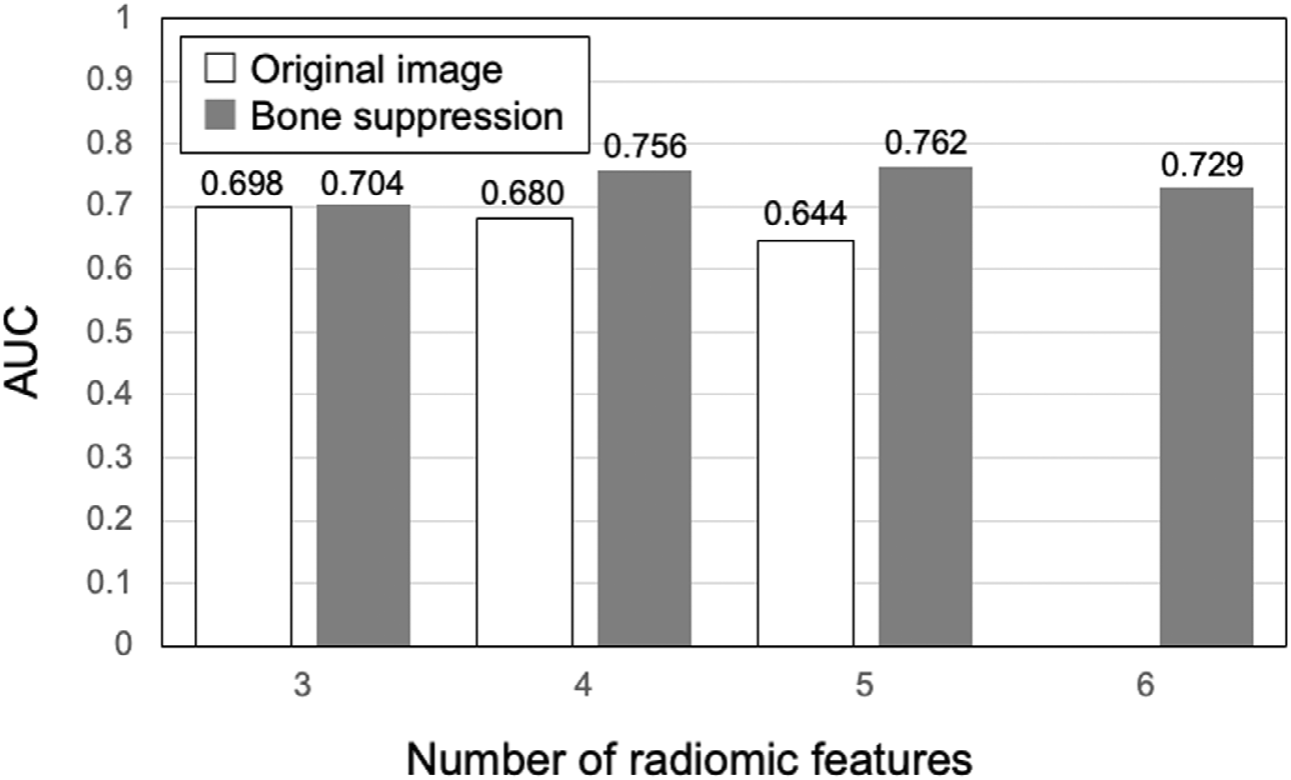
Relationship between the number of selected radiomic features and the area under the receiver‐operating characteristic curve (AUC) for the prediction of mortality risk when a linear discriminant analysis is used.

Figure 3 shows the relationship between the number of radiomic features selected by Lasso and the AUCs. This result was obtained by comparing the discriminative performance of the LDA with or without the bone‐suppression technique. In the absence of bone suppression, six radiomic features were not selected by Lasso. With bone suppression, the highest AUC was 0.762 when five radiomic features were used. Without bone suppression, the highest AUC value was 0.698 when three radiomic features were used. Since the highest AUC on average was obtained when four radiomic features were used, processing was performed using four radiomic features. Table 1 shows the four radiomic features selected by Lasso with and without bone suppression. It can be seen that texture features obtained by the density co‐occurrence matrix were mainly selected. S(5,0) shows that the co‐occurrence matrix was calculated with a distance of 5 pixels on the X‐axis and 0 pixels on the Y‐axis. Haar function is employed as a wavelet, and LH represents a low‐pass filter in the X‐axis direction and a high‐pass filter in the Y‐axis direction. For detailed definitions of these radiomic features, refer to.^29^

**Table 1:** Radiomic features selected by the least absolute shrinkage and selection operator technique with and without bone suppression. For detailed definitions of these radiomic features, refer to http://www.eletel.p.lodz.pl/programy/mazda/.^29^

Image	#	Feature	Lung	Category	Description
Original image	#1	S(5,0)DifVarnc	Left	Texture feature Co‐occurrence matrix	Difference variance S(5,0) is the between‐pixel distance
	#2	S(0,1)SumVarnc	Right	Texture feature Co‐occurrence matrix	Sum Variance S(0,1) is the between‐pixel distance
	#3	S(0,3)SumEntrp	Right	Texture feature Co‐occurrence matrix	Sum Entropy S(0,3) is the between‐pixel distance
	#4	S(0,4)SumEntrp	Right	Texture feature Co‐occurrence matrix	Sum Entropy S(0,4) is the between‐pixel distance
Bone Suppression	#1	S(5,0)DifVarnc	Left	Texture Co‐occurrence matrix	Difference variance S(5,0) is the between‐pixel distance
	#2	WavEnLH_s‐7	Left	Resolution Haar Wavelet	Wavelet energy (frequency band: LH, 7rd scale)
	#3	S(0,1)SumOfSqs	Right	Texture Co‐occurrence matrix	Sum of squares S(0,1) is the between‐pixel distance
	#4	S(0,1)SumVarnc	Right	Texture Co‐occurrence matrix	Sum Variance S(0,1) is the between‐pixel distance

**Figure 4:**
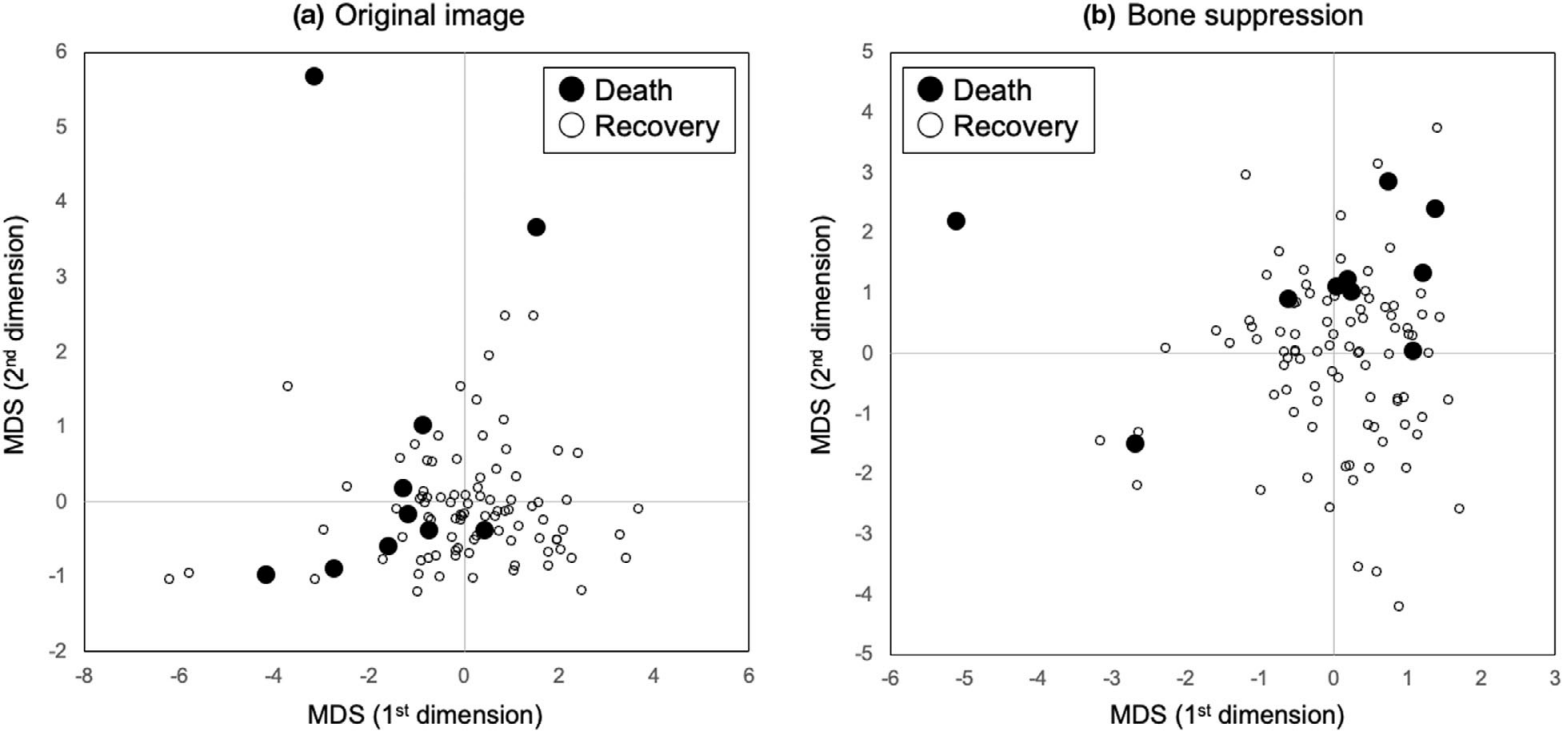
Multidimensional scaling (MDS) outputs using four radiomic features with and without bone suppression.

Figure 4 shows the MDS results using the four radiomic features with and without bone suppression. Since the radiomic features were normalised, and the number of recovery cases was larger than the number of death cases, the recovery cases were distributed around the origin. In addition, death cases were distributed away from the origin and did not tend to be distributed in a specific direction. Without bone suppression, there was a tendency for the overlap between the death and recovery cases to be large.

**Figure 5:**
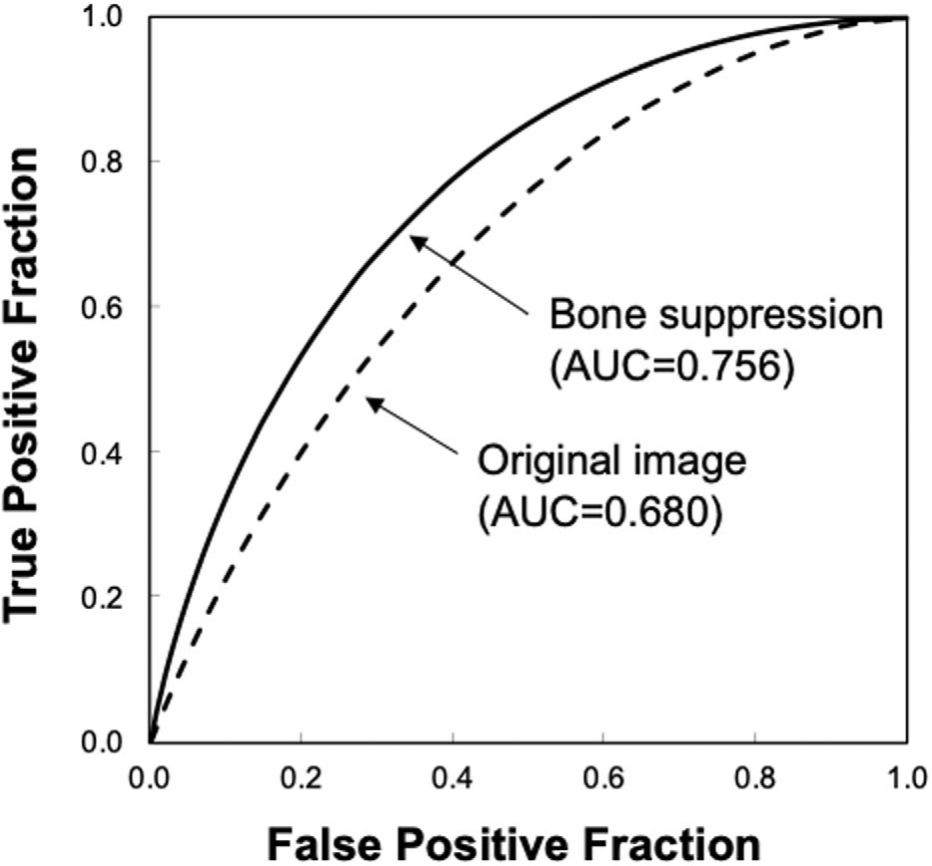
Results of the receiver‐operating characteristic analysis for distinguishing between death and recovery cases when a linear discriminant analysis is used. AUC—area under the receiver‐operating characteristic curve.

**Figure 6:**
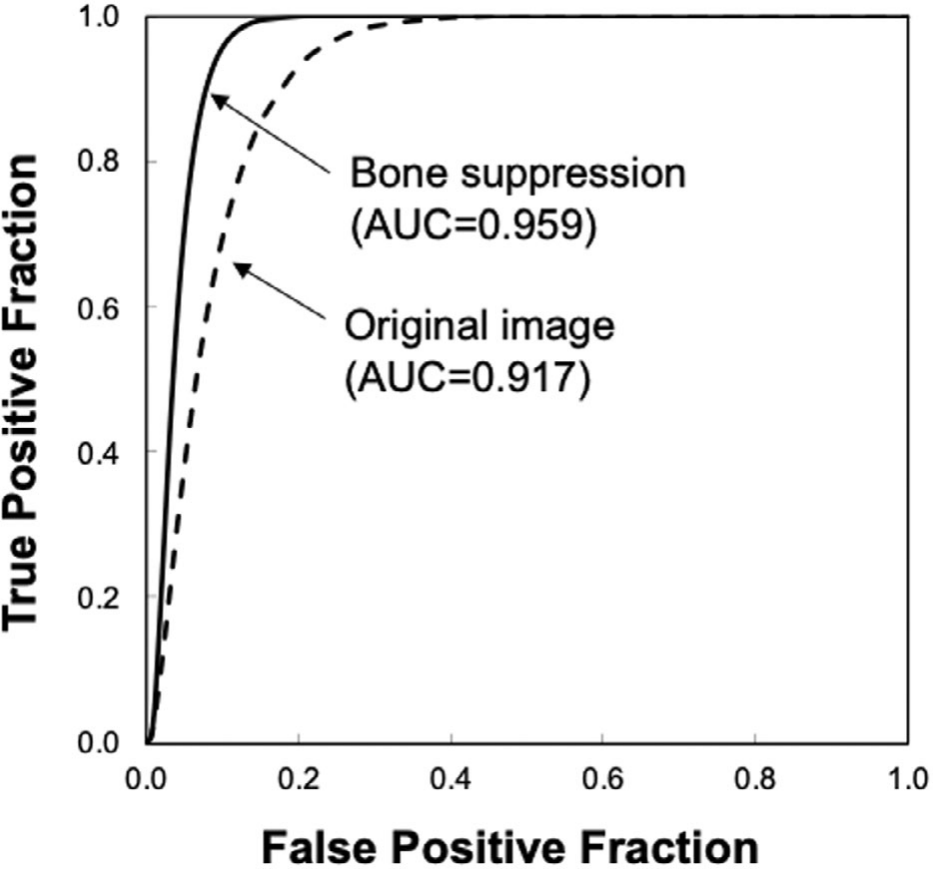
Results of the receiver‐operating characteristic analysis for distinguishing between death and recovery cases when a support vector machine is used.

Figure 5 shows the ROC curves with and without bone suppression for predicting death and recovery cases. Because the two ROC curves did not intersect, the discriminative performance could be directly compared using AUC values. The discriminative performance was higher when bone suppression was used, with AUCs of 0.756 ± 0.149 and 0.680 ± 0.171. Figure 6 shows the results obtained using the SVM. These parameters were empirically determined. Similar to the LDA results, the discriminative performance of the SVM with bone suppression was higher than without bone suppression, with AUCs of 0.959 ± 0.037 and 0.917 ± 0.060. As shown in Figure 3, death cases were not distributed in a specific direction from the origin; therefore, it was difficult to distinguish them on a hyperplane. Because SVM generates a complex discriminant boundary, SVM achieved better discriminative performance than LDA. When an SVM was used, the sensitivity for predicting death was 90.0% (9/10), and the specificity was 95.6% (86/90).

## Discussion

Using LDA and SVM evaluations, the discriminative performance for identifying death versus recovery cases was improved with bone suppression. The analysis of chest radiographs can be difficult because of the overlap between bone components and the abnormal patterns of COVID‐19. However, it is thought that these abnormal patterns can be analysed more easily when bone‐suppression techniques are applied. Therefore, bone suppression is considered useful as a pre‐processing method because it is an image processing and does not require the use of energy subtraction.

In addition, if LDA provides high discriminative performance, it means that there is a simple relationship between radiomic features and the COVID‐19 patients in danger of death. Therefore, radiologists can use the imaging findings that match the radiomic features for predicting the mortality risk of COVID‐19. However, the discriminative performance of LDA was not high, even when bone suppression was used. Therefore, the relationship between imaging findings and mortality risk is not simple, and it may be difficult for radiologists to use radiomics features as imaging biomarkers for this prediction. It should be noted that LDA was employed to investigate whether there is a simple relation between radiomic features and mortality risk, and LDA was not used for predicting the mortality risk of COVID‐19 in this study. Predictive models have a trade‐off between predictive accuracy and interpretability. Conventional statistical models have high interpretability due to their simple design. A model with low interpretability is called black box. However, it is ideal to have both high predictive accuracy and high interpretability.

When SVM was used, mortality risk could be predicted with high accuracy, suggesting that AI can identify complex relationships that are difficult for humans to interpret from imaging data easily. By showing SVM output to a doctor, it may be possible to achieve augmented intelligence that expands a doctor's knowledge and helps him or her make an accurate diagnosis. This new concept is similar to that of traditional CAD. In CAD, for the detection of the lesion, the radiologist reconfirms the image based on the output of the computer and determines whether the lesion exists. By evaluating the computer results, the radiologist can easily notice his or her overlook of the lesion when the computer detected the lesion accurately, and the radiologist can easily recognise the false detection when the computer detected normal tissue as a lesion. Therefore, the synergistic effect between the radiologist and the computer improves the accuracy of the diagnosis.^1^ However, since radiomics estimates the future condition from a current image, it is difficult for radiologists to adapt to this technology. Hence, it is necessary to clarify what type of information should be provided to the doctor to improve the predictive accuracy. Since it is much more difficult to develop an explainable AI system than a CAD system for the detection of diseases, this should be pursued in future research.

In current studies on COVID‐19, there is no distinction between approaches for detection and prognostic prediction. Deep learning is also used for prognostic prediction.^16^, ^18^, ^19^ However, in the AI system for prognostic prediction, it is important to develop a method that explains the computer results for the doctor. From this point of view, un‐box deep learning approaches have been developed. However, an observation study is needed to verify what information helps doctors make clinical decisions and improve predictive performance.

The main limitation of this study was the small number of death cases. Since the radiomic features selected by Lasso depended on the cases used in this study, it will be necessary to increase the number of cases to improve the reliability of this system in the future.

## Conclusions

In this study, we demonstrated it is possible to predict COVID‐19 patients in danger of death using radiomic features obtained from portable chest radiographs. We also confirmed that a bone‐suppression technique was effective. In the future, we will further explore the utility of using portable chest radiographs for this purpose by increasing the number of cases and improving the reliability of the system.

## Conflict of Interest

H. Minami is an employee of Konica Minolta, Inc.

## Ethics

We used data from a public database in this study. The Kumamoto University Institutional Review Board of allowed us to use data from those cases.
